# Incorporating Linguistic Knowledge for Learning Distributed Word Representations

**DOI:** 10.1371/journal.pone.0118437

**Published:** 2015-04-13

**Authors:** Yan Wang, Zhiyuan Liu, Maosong Sun

**Affiliations:** 1 State Key Laboratory of Intelligent Technology and Systems, Tsinghua National Laboratory for Information Science and Technology, Department of Computer Science and Technology, Tsinghua University, Beijing, China; 2 Jiangsu Collaborative Innovation Center for Language Competence, Jiangsu, China

## Abstract

Combined with neural language models, distributed word representations achieve significant advantages in computational linguistics and text mining. Most existing models estimate distributed word vectors from large-scale data in an unsupervised fashion, which, however, do not take rich linguistic knowledge into consideration. Linguistic knowledge can be represented as either link-based knowledge or preference-based knowledge, and we propose knowledge regularized word representation models (KRWR) to incorporate these prior knowledge for learning distributed word representations. Experiment results demonstrate that our estimated word representation achieves better performance in task of semantic relatedness ranking. This indicates that our methods can efficiently encode both prior knowledge from knowledge bases and statistical knowledge from large-scale text corpora into a unified word representation model, which will benefit many tasks in text mining.

## Introduction

The performance of text mining is heavily dependent on word representation. The most widely used methods of word representation are vector space models (VSM) [1], which represent word meanings with vectors, with each dimension corresponding to semantic or syntactic information of words. VSM can be easily used to conduct similarity measures by computing distances between vectors, and thus are widely adopted in various applications such as information retrieval, text classification and question answering.

The basic idea of learning word representations is assuming contextual information of words provides a good clue to word meaning, and similar words tend to share similar distributions of contextual information. For instance, distributional semantic models (DSM) [2] use vectors to record contexts (e.g., co-occurring words) in which target words appear in a large corpus. It has long been known that simple co-occurrence counts do not work well for DSM. Techniques such as reweighting, smoothing and dimension reduction have been proposed to enhance performance [2]. However, these optimization techniques require heavily manual tuning. Moreover, DSM is non-trivial to be extended to higher level representation of sentences or documents.

By contrast, prediction-based methods are developed to build word representation. These methods estimate word vectors so as to maximize the predictive probability of the contexts when observing a target word in the corpus. Among these methods, neural language models (NLM) [3] are the most attractive due to their impressive characteristics.

- The dimension of word vectors in NLM is relatively lower (usually ranging from 10 to 2000). Its capability of representation grows at exponential speed with the increase of vector dimension. Since word meanings are represented as a vector of real values, it is named as *distributed word representation* or *word embedding*.
- The representation in continuous vector space brings easy measurement of similarity between two words, and complicated smoothing techniques are not necessary.
- Both syntactic and semantic properties of words are encoded into the unified word representations from large-scale corpora, which can be easily adopted by multi-task applications.

With these advantages, distributed word representation has shown its power with promising performance in many applications [4, 5].

A large-scale corpus is required for sufficient estimation of word vectors in distributed representation [3]. In the big data era, computational efficiency is becoming increasingly crucial, and many distributed representation methods based on neural networks heavily suffer from high computational complexity. To address the computational efficiency issue, recently two simple and powerful models have been proposed for learning distributed representation [6]: Continuous Bag-of-Words Model (CBOW) and Continuous Skip-gram Model. By discarding non-linear hidden layer, both the models manage to learn from large-scale corpora efficiently.

Most existing methods for distributed word representation are unsupervised and only learn from text corpora. As a matter of fact, people have constructed a variety of knowledge bases about words and languages. Seeing that tremendous linguistic knowledge is ready in these knowledge bases, it is fairly intuitive for us to consider incorporating the prior knowledge in word representation learning from text corpora.

We can gain great advantages by incorporating external prior knowledge into word representation learning.

- Prior knowledge provides more useful information from knowledge bases beyond statistics from corpora. For example, after knowing that both *car* and *automobile* refer to the same meaning in the real world, we conclude that they are synonyms. Although these two words share the same meaning, their context might not be so similar because they are often used in different linguistic styles. Hence, the prior knowledge may, to some extent, fix the bias of word vectors learned from corpora. The bias may be caused for various reasons, such as the domains of corpora, or statistical insufficiency of those low-frequency words.
- Many specific domains, such as some scientific research areas, usually maintain rich domain knowledge bases. When entering these domains, especially when domain-specific corpora are not sufficient enough compared to general corpora, the domain-specific prior knowledge will provide essential help to learn good domain-specific word representation. It will be of great significance for domain adaptation of text mining applications.

Moreover, knowledge bases are usually constructed manually and thus are more reliable than statistical methods. Various types of knowledge bases have been manually developed by human experts. Here are some typical ones:

- **WordNet** [7] is a lexical knowledge base for English, which groups English words into set of synonyms named as *synsets*. For example, the above-mentioned *car* and *automobile* lie in the same synset. Besides, WordNet also records semantic relations between these synsets, such as part-of and hypernym relations.
- **Word Association Network (WAN)** [8] is a dictionary produced from a word game. In this game, a person is shown a randomly-picked word, and is asked to write those words that he/she arises in mind. WAN records the association relations from given words to their associated words. For example, people may tend to associate with *Christmas* when given the word *gift*.
- **PPDB** [9] is a database of paraphrase rules extracted automatically by comparing millions of paraphrase pairs. This database classifies paraphrase rules into four classes including lexical rules, one-to-many rules, phrasal rules and syntactic rules. Each of the lexical rules contains two words (*w*_*i*_,*w*_*j*_) indicating that *w*_*j*_ can replace *w*_*i*_ when paraphrasing. Almost for each lexical rule (*w*_*i*_,*w*_*j*_), there is an inverse rule (*w*_*j*_,*w*_*i*_). So we can extract many word pairs that share the same semantic meanings.

Knowledge bases usually contain complicated and heterogenous information. It is unnecessary and impossible to take all of them into consideration. In this paper, we consider the semantic relevance information between words provided by knowledge bases for word representation learning. The semantic relevance knowledge can be represented as either link-based knowledge or preference-based knowledge:

- **Link-based Knowledge.** We can use links to represent semantic relevance between words, i.e., a link between two words indicating they are relevant and no link indicating irrelevant. For example, we can transform either synsets in WordNet or associations in WAN into link-based knowledge. Moreover, we can also assign weights to links as relatedness between words.
- **Preference-based Knowledge.** The preference-based knowledge does not assign absolute relatedness score between words, and only records preference ranks according to relatedness. Take WAN for example, for a given word, we can rank associated words according to the number of people mentioning them, and provide preference-based knowledge of word pairs.

Link-based knowledge and preference-based knowledge are two distinct types of word knowledge, and require different techniques to incorporate into word representation.

In some cases, link-based knowledge can be transformed into preference-based knowledge. For example, we could simply rank word pairs according to their relatedness scores in link-based knowledge, or pick any linked word pairs against any unlinked pairs to build preference-based knowledge. It is usually difficult for people to objectively and sophisticatedly determine the absolute relatedness scores of word pairs in isolation. People may concern more about the preference among word pairs. Hence, it may be more reliable to consider preference-based knowledge instead of weighted link-based knowledge.

The transformation apparently does not work all the time. Take synsets in WordNet for example, we only know the words in a synset are relevant to each other, but do not know which pairs are more relevant than another. Moreover, link-based knowledge does provide relevant word pairs, but does not indicate all remaining pairs are irrelevant. Hence, we cannot simply regard linked word pairs preferred thank all unlinked pairs.

In this paper, we take prior word knowledge into distributed word representation, and propose a unified framework named as **Knowledge Regularized Word Representation (KRWR)**. In principle, KRWR works for all methods of distributed word representation, but since it is impossible to investigate the effectiveness of all representation models, we only take CBOW as the typical model for study because of its efficiency on big data and recent popularity. Experiments on real-world data sets demonstrate the learned word vectors can successfully encode prior knowledge, which will greatly benefit a collection of text mining applications.

## CBOW Model

The architecture of Continuous Bag-of-Words Model proposed in [6] is similar to the feed-forward neural-network language model (NNLM). It is named as a bag-of-words model because all words within the context window are projected to the same position in projection layer, without considering the order of words. The non-linear hidden layer in NNLM is removed to accelerate training process.

**Fig 1:**
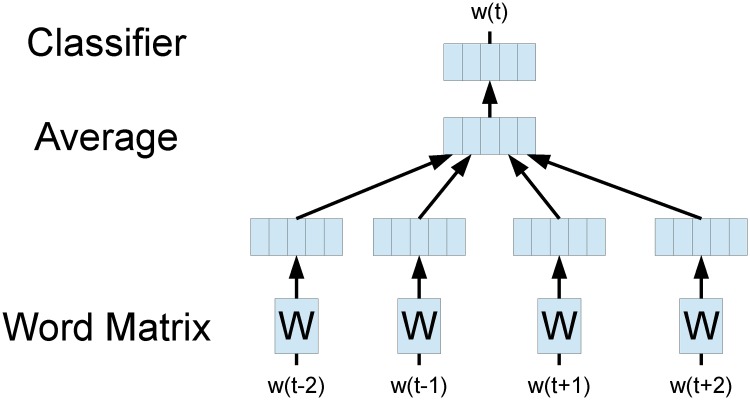
CBOW model.

In CBOW model, each word corresponds to a unique vector, represented as a column in a word matrix *W* ∈ ℝ^*K*×*V*^, where *K* is the dimension of a word vector, and *V* is the size of word vocabulary. Given each window in a sentence, the sum of contextual word vectors is used as features to predict the target word. The framework of CBOW model is demonstrated in Fig. 1.

Formally, given a sequence of words, *D* = (*w*_1_,…,*w*_*T*_), the objective function of the CBOW model is to maximize the average log-likelihood,

$$L\left(D\right)=\sum_{t<|T|}\log\mathrm{Pr}\left(w_{t}|w_{t-s},\ldots ,w_{t-1},w_{t+1},\ldots ,w_{t+s}\right)$$ (1)

where the windows size is 2*s*+1. The prediction task is a typical multi-class classification problem.

We denote the context (*w*_*t*−*s*_,…,*w*_*t*−1_,*w*_*t*+1_,…,*w*_*t*+*s*_) as *w*_*c*_. The prediction probability can be typically defined with a *softmax* fashion as follows,

$$\mathrm{Pr}\left(w_{t}|w_{c}\right)=\frac{\exp\left(g(w_{t},w_{c})\right)}{\sum_{i}\exp\left(g(w_{i},w_{c})\right)}$$ (2)

where *g*(*w*_*t*_,*w*_*c*_) indicates the un-normalized log-probability for each output word *w*_*t*_. In CBOW model, the probability is simply defined as

$$g\left(w_{t},w_{c}\right)={\vec{w}}_{t}\cdot {\vec{w}}_{c}$$ (3)

where $\vec{w}$ indicates the distributed embedding vector for a word *w*, and the operator ⋅ is the dot product of two vectors. For implementation, *hierarchical softmax* [10] is adopted to softmax for training, and we follow the work in [6] and build a binary Huffman tree as the structure of hierarchical softmax. The training complexity is *T*×*K*+*K*×log(*V*), where *T* is the window size, *K* is the dimension of word vectors and *V* is the size of vocabulary.

In this paper, we present our method based on the CBOW model, i.e. Knowledge Regularized CBOW (KCBOW), and implement based on an open source project *word2vec* [6, 11] (https://code.google.com/p/word2vec/). Note that, our work can be easily extended to other methods of distributed word representation.

## Prior Knowledge Construction in KCBOW

As mentioned in the introduction section, from knowledge bases including but not limited to WordNet, WAN and PPDB, we can extract either link-based knowledge or preference-based knowledge. Link-based knowledge and preference-based knowledge are related to each other but not identical. We take WordNet and WAN for example, to demonstrate the construction of prior knowledge.

### 2.1 Link-based Knowledge Construction

**Fig 2:**
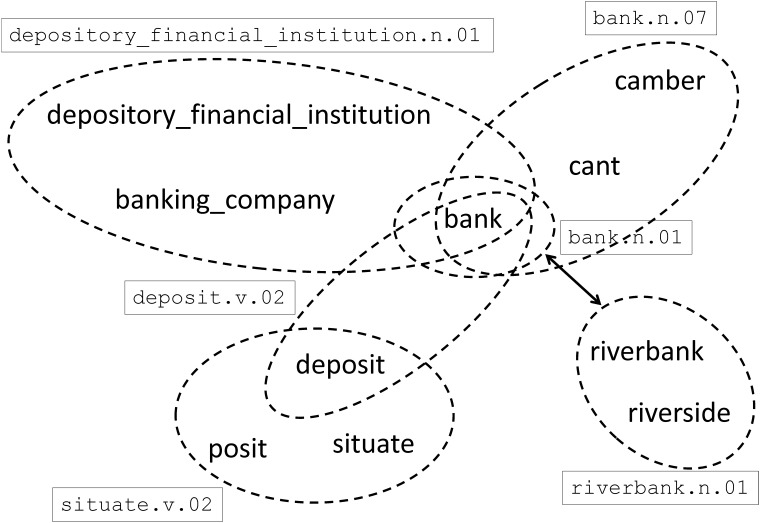
A small miniature of topology in WordNet. The word *bank* is related to many other words by synsets (represented with dashed circles) and hyponym/hypernym relationship (represented with arrow).

In WordNet, each synset indicates a unique word sense, usually including several words with identical or similar semantic meanings. There are also multiple types of relations between synsets, such as part-of and hypernym. In Fig. 2, we demonstrate a small miniature of topology in WordNet, by taking the word *bank* as our focus. From the figure, we observe that the word *bank*, as a polysemous word, appears in multiple synsets (represented with dashed circles) and connects them together. Meanwhile, the relation hyponym/hypernym between synsets (represented with arrow) also connects synsets together.

**Fig 3:**
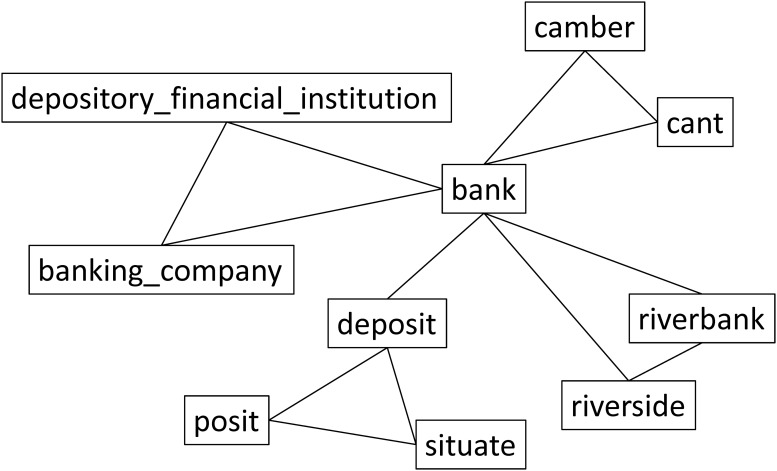
Link-based knowledge is constructed according to the topology as shown in Fig. 2.

Based on synsets and relations, we can build a word graph and construct link-based knowledge as shown in Fig. 3. In this graph, two connected words are considered to be semantically related with each other.

Formally, we construct a prior knowledge matrix *P* ∈ ℝ^*V*×*V*^, in which *P*_*ij*_ = *p*(*w*_*i*_,*w*_*j*_) is the relatedness between the *i*th word and the *j*th word in vocabulary. According to Fig. 3, we can simply set *p*(*w*,*v*) = 1 if there is a link between the word *w* and *w*, and otherwise *p*(*w*,*v*) = 0. We can also employ other sophisticated measures to compute semantic relatedness between words according to the topology of WordNet, such as the shortest-path method, et al. However, these measures are not guaranteed to be always correct, and hence in this paper we select the above-mentioned simple version.

### 2.2 Preference-based Knowledge Construction

Preference-based knowledge provides a different perspective as compared to link-based knowledge. The preference-based knowledge is essential for the following two reasons: (1) Under some circumstances, link-based knowledge is not available or sufficient. (2) Sometimes we may care more about the preference order by relatedness of word pairs, instead of the absolute relatedness scores of word pairs in isolation. Take WAN for example, each word may be associated to tens or hundreds of words in different number of times. We cannot well distinguish which word pairs should be linked together and which are not. In this case, it is more appropriate for us to construct preference-based knowledge according to the number of association times instead of link-based knowledge.

We construct preference-based knowledge *P* from WAN as follow. For a word *w*_*s*_ in a WAN, suppose the associated word list is {(*w*_1_,*c*_1_),…, (*w*_*L*_,*c*_*L*_)}, where *c*_*i*_ is the association count indicating how many people associates the word *w*_*i*_ given the word *w*_*s*_, and *L* is the list size. For each two words *w*_*i*_ and *w*_*j*_ in this list, if *c*_*i*_ > *c*_*j*_, we can get the preference-based knowledge *w*_*i*_ ≻ *w*_*j*_, meaning that the word *w*_*c*_ prefers to be more related to *w*_*i*_ compared to *w*_*j*_. We can thus get a preference-based knowledge fact (*w*_*s*_,*w*_*i*_,*w*_*j*_) ∈ *P*, indicating *w*_*i*_ ≻ *w*_*j*_ with respect to the target word *w*_*s*_. Note that, if two words are equally associated to the target word, we will not build preference-based knowledge for them. We exclude triples in which two words are equally associated to the target word.

Take the word *bank* for example, in WAN its top-3 association words are {(*money*, 115), (*account*, 5), (*robber*, 5)}. According to the list, we can build the following preference-based knowledge (*bank*, *money*, *account*) and (*bank*, *money*, *robber*).

The idea of preference-based knowledge is, to some extent, related to the framework of learning to rank in information retrieval [12, 13]. Link-based knowledge and preference-based knowledge can transfer to each other: (1) For any *p*(*w*_*s*_,*w*_*i*_) > *p*(*w*_*s*_,*w*_*j*_) in link-based knowledge, we can construct a preference-based knowledge fact (*w*_*s*_,*w*_*i*_,*w*_*j*_). (2) For any (*w*_*s*_,*w*_*i*_,*w*_*j*_) in preference-based knowledge, we can simply construct link-based knowledge *p*(*w*_*s*_,*w*_*i*_) = *m* and *p*(*w*_*s*_,*w*_*j*_) = *n* guaranteeing *m* > *n*. However, the transformation usually introduces noise and loses information. For example, the transformation in Type (1) may lose the scoring information of *p*(*w*_*s*_,*w*_*i*_) and *p*(*w*_*s*_,*w*_*j*_), and the setting of *m*,*n* in Type (2) will introduce much noise.

In summary, link-based knowledge and preference-based knowledge provide us distinct perspectives to prior knowledge, and in the following section, we will show how to learn distributed word representation with the prior knowledge.

## Learning Word Representation with Prior Knowledge

We propose a new framework for learning distributed word representation by incorporating prior knowledge as a regularizer. The idea of the regularization is straightforward: words which are semantically relevant to each other should have similar vectors. Formally, we define a regularized likelihood as follows,

O(D,P)=(1-λ)L(D)+λR(D,P), (4)

where *L*(*D*) is the log-likelihood of the word sequence *D*, and *R*(*D*,*P*) is a harmonic regularizer defined on prior knowledge *P*, and *λ* is the harmonic factor, with a range [0, 1]. The learning algorithm will aim to maximize *O*(*D*,*P*). When *λ* = 0, optimizing *O*(*D*,*P*) is identical to maximizing *L*(*D*), and when *λ* = 1, the optimization of *O*(*D*,*P*) will only depend on prior knowledge.

### 3.1 Learning with Link-based Knowledge

For link-based knowledge, we define *R*(*D*,*P*) as follows,

$$R\left(D,P\right)=\sum_{w,v\in V}p\left(w,v\right)\log r\left(w,v\right),$$ (5)

where *p*(*w*,*v*) indicates the prior relatedness between the words *w* and *v*, and *r*(*w*,*v*) is the relatedness measured with distributed representation vectors of *w* and *v*. The relatedness *r*(*w*,*v*) can be measured with various methods. For example, we can define *r*(*w*,*v*) with softmax probability,

$$r\left(w,v\right)=\frac{\exp\left(g(w,v)\right)}{\sum_{v^{'}}\exp\left(g(w,v^{'})\right)}.$$ (6)

In this case, *r*(*w*,*v*) ≠ *r*(*v*,*w*), and thus we will consider the cases in both sides in Eq. (5). In the following sections, this regularizer is referred to as Softmax Probability Regularizer (SPR).

We can also adopt Euclidean distance to measure the relatedness, i.e.,

$$r\left(w,v\right)=\exp\left(-∥\vec{w}-\vec{v}{∥}_{2}^{2}\right).$$ (7)

We refer to this regularizer as Euclidean Regularizer (ER). Note that, Euclidean distance is not compatible with hierarchical softmax used for optimizing *L*(*D*).

The prior relatedness between *w* and *v* may be asymmetric, i.e., *p*(*w*,*v*) ≠ *p*(*v*,*w*); meanwhile *r*(*w*,*v*) ≠ *r*(*v*,*w*). Hence, we formalize *R*(*D*,*P*) as follows,

$$R\left(D,P\right)=\frac{\lambda }{2}\sum_{w,v\in V}\left(p(w,v)\log r(w,v)+p(v,w)\log r(v,w)\right).$$

Take the format of SPR for example, we finally have the objective function as follows,

$$O\left(D,P\right)=\left(1-\lambda \right)\sum_{t<|T|}\log\frac{\exp\left({\vec{w}}_{t}\cdot {\vec{w}}_{c}\right)}{\sum_{i}\exp\left({\vec{w}}_{i}\cdot {\vec{w}}_{c}\right)}+\frac{\lambda }{2}\sum_{w,v\in V}\left(p\left(w,v\right)\log\frac{\exp\left(\vec{w}\cdot \vec{v}\right)}{\sum_{v^{'}}\exp\left(\vec{w}\cdot {\vec{v}}^{'}\right)}+p\left(v,w\right)\log\frac{\exp\left(\vec{w}\cdot \vec{v}\right)}{\sum_{w^{'}}\exp\left(\vec{w^{'}}\cdot \vec{v}\right)}\right).$$ (8)

### 3.2 Learning with Preference-based Knowledge

With preference-based knowledge, we consider the preference relations between two word pairs. Given a preference fact (*w*_*s*_,*w*_*i*_,*w*_*j*_) ∈ *P*, we want to make sure *r*(*w*_*s*_,*w*_*i*_) > *r*(*w*_*s*_,*w*_*j*_). That is, we want to make sure *w*_*s*_ is more related with *w*_*i*_ as compared to *w*_*j*_. In this paper, we propose two methods to define *R*(*D*,*P*) for preference-based knowledge.

It is straightforward for us to model preference-based knowledge using a margin-based ranking criterion, defined as follows,

$$R\left(D,P\right)=\sum_{(w_{s},w_{i},w_{j})\in P}{\left[r\left(w_{s},w_{i}\right)-r\left(w_{s},w_{j}\right)-\gamma \right]}_{-},$$ (9)

where *r*(*w*,*v*) is the semantic relatedness between the words *w* and *v* measured with distributed representation vectors of *w* and *v*, [*x*]_−_ denotes the negative part of *x*, and *γ* is a hyper-parameter indicating the margin. The regularizer is named as Margin Regularizer (MR). As mentioned in the last section, *r*(*w*,*v*) can be calculated with either softmax probability or Euclidean relatedness. In empirical experiments, we find that the performance of MR with softmax probability is poor as compared to the other methods. Hence, we only show the results of using Euclidean relatedness for MR.

Alternatively, inspired by the idea of Negative Sampling [11], for a preference-based knowledge fact (*w*_*s*_,*w*_*i*_,*w*_*j*_) ∈ *P*, we can also distinguish the more related word *w*_*i*_ from *w*_*j*_ using logistic regression, and thus define *R*(*D*,*P*) as follows,

$$R\left(D,P\right)=\sum_{(w_{s},w_{i},w_{j})\in P}\log\sigma \left({\vec{w}}_{s}\cdot {\vec{w}}_{i}\right)+\log\sigma \left(-{\vec{w}}_{s}\cdot {\vec{w}}_{j}\right),$$ (10)

where σ(x)=1/(1+exp(−x)) is the sigmoid function. Following negative sampling, we use inner product between two word vectors to indicate their relatedness. The regularization is named as Negative Sampling Regularizer (NSR).

There are two differences between MR and NSR: (1) They use different methods to measure semantic relatedness between two words, MR with Euclidean distance and NSR with inner product. (2) MR set a margin between two word pairs, which enhances the discrimination ability of MR as compared to NSR. Meanwhile, the performance of MR is sensitive to the setting of the margin.

Similar to the modeling method of link-based knowledge, we can incorporate *R*(*D*,*P*) with existing representation modeling method *L*(*D*) into *O*(*D*,*P*). In the following section, we will introduce the methods for parameter estimation.

### 3.3 Parameter Estimation

We learn KCBOW models using stochastic gradient descent (SGD). In *O*(*D*,*P*), *L*(*D*) and *R*(*D*,*P*) are learned with different data sets, but aim at learning a unified distributed word representation. For the *L*(*D*) part, we apply the idea in [6, 11] and adopt both hierarchical softmax and negative sampling for optimization. For *R*(*D*,*P*), we propose two schemes for learning: joint optimization and post optimization.

**Joint Optimization (JO).** In *word2vec*, word representation is learned using the asynchronous version of stochastic gradient descent (ASGD), with multiple threads, using different training data and updating shared word vectors. It is thus straightforward for us to perform joint optimization of *L*(*D*) and *R*(*D*,*P*) with multiple threads. Each thread is assigned to optimize either *L*(*D*) or *R*(*D*,*P*) with thread-specific data, and update shared word vectors.

**Post Optimization (PO).** As mentioned in the previous section, Euclidean distance is not compatible with hierarchical softmax used for optimizing *L*(*D*), and thus cannot perform joint optimization. We hence propose post optimization for ER and MR. That is, after the learning of *L*(*D*) or the joint optimization of *O*(*D*,*P*), we take the learned word representation as a new starting point, and begin to optimize *R*(*D*,*P*) according to ER (Eq. (7)) and MR (Eq. (9)). However, this may cause overfitting. Therefore, we may modify Eq. (7) and Eq. (9) to avoid overfitting, defined as follows,

$$R^{'}\left(D,P\right)=R\left(D,P\right)-\delta \left(∥{\vec{w}}_{s}-{\vec{w}}_{s}^{'}∥+∥{\vec{w}}_{i}-{\vec{w}}_{i}^{'}∥+∥{\vec{w}}_{j}-{\vec{w}}_{j}^{'}∥\right)$$ (11)

where ${\vec{w}}_{s}^{\prime }$, ${\vec{w}}_{i}^{\prime }$ and ${\vec{w}}_{j}^{\prime }$ are original word vectors learned before post optimization, and *δ* ranges from 0.0 to 1.0. By adding the second formula in Eq. (11) as a penalty, we prevent the optimization from moving word vectors too far away from original learned vectors. Obviously, other optimization methods for *R*(*D*,*P*) can also be used in post optimization, by simply adding the penalty formula.

Note that, for SPR and ER of link-based knowledge and MR of preference-based knowledge, we can perform both joint optimization and post optimization, whereas for NSR of preference-based knowledge, we can only perform post optimization.

Model initialization plays an important role in deep learning. In joint optimization, we initialize all dimensions of vectors with random small real numbers. In post optimization, we first randomly initialize the vectors before pre-training CBOW and then take these pre-trained vectors to initialize the regularization process.

## Experiments and Analysis

In this section, we first introduce the datasets and construction of prior knowledge, then describe the evaluation tasks, metrics and results, and analyze the influence of some parameters. At the end of this section, we evaluate our models with the task of semantic relatedness ranking to demonstrate that, incorporating prior knowledge is critical to improve the quality of distributed word representation.

### 4.1 Datasets

We select July 2013 snapshot of Wikipedia (http://dumps.wikimedia.org/enwiki/20130708/) and extract all articles with Wikipedia Extractor (http://medialab.di.unipi.it/wiki/Wikipedia_Extractor) as training corpora of distributed word representation. The vocabulary consists of about 185 thousand words which appear more than 100 times in the corpora. There are 1.36 billion tokens in the training corpora in total. All tokens are transformed to lower case. During the training process, we ignore those tokens that are not included in the vocabulary.

In our experiments, we select three datasets to construct link-based knowledge and preference-based knowledge, including WordNet, WAN and PPDB.

WordNet (http://wordnet.princeton.edu/) [7] is a large English lexical knowledge base. In WordNet, nouns, verbs, adjectives and adverbs are manually grouped into sets of synonyms (synsets), with each expressing a distinct concept. Synsets are connected with each other according to their semantic and lexical relations. WordNet contains about 150 thousand words organized in about 120 thousand synsets. In this paper, we extract link knowledge from WordNet version 2.1 with the help of JWI (http://projects.csail.mit.edu/jwi/). We consider two relations in WordNet to construct link-based knowledge: (1) It is straightforward that, for each pair of words in the same synset, they are linked since they share the same word sense. However, there are usually not many words in each synset on average. It is not sufficient to construct link-based knowledge using only synsets. (2) We also consider relations between synsets to construct link-based knowledge. To be specific, if synset *S*_*i*_ and *S*_*j*_ are linked by a certain kind of relation, then we add links between all pairs (*w*,*v*) if and only if *w* ∈ *S*_*i*_ and *v* ∈ *S*_*j*_.

The University of South Florida Free Association Norms [8] (http://w3.usf.edu/FreeAssociation/) is a large WAN database, involving more than 6,000 participants. 5,019 words are selected as cues and three quarters of a million responses are collected. This database offers 72,000 word pairs and a sample of it is shown in Table 1. #G is the size of participant group given the cue word, and #P is number of participants who produce the target. For example, given cue word *bank*, 115 out of 144 participants produce the response of *money*. This dataset can construct both link-based knowledge and preference-based knowledge. We select triples {(*w*_*s*_,*w*_*i*_,*w*_*j*_)} that meet the criteria (#*P*_*s*_(*i*)−#*P*_*s*_(*j*))/#*G* > 0.02 to construct knowledge.

**Table 1:** Sample of University of South Florida Free Association Norms.

Cue	Target	#G	#P
bank	money	144	115
bank	account	144	5
bank	robber	144	5
bank	teller	144	4
bank	loan	144	2
bank	vault	144	2

PPDB [9] (http://www.cis.upenn.edu/~ccb/ppdb/) is a paragraph dataset with over 220 million English paraphrase pairs, consisting of 73 million phrasal and 8 million lexical paraphrases. PPDB is organized in six packages ranging from S (Small) to XXXL (3-eXtreme Large), with different trade-off between precision and coverage. We choose the lexical rules of XL, XXL and XXXL packages as link-based knowledge. There are overlaps among the three packages.

For all datasets, we abandon the links and triples containing words not included in our vocabulary. For each of the datasets, we randomly select around 1/25 of links or preference triples to construct testing sets, and the rest form training sets. The actual number of links in each set is listed in Table 2.

**Table 2:** Number of Links in Different Datasets.

Dataset	Training Set (thousand)	Testing Set (thousand)
XL	125	5
XXL	598	30
XXXL	2640	100
WN	2000	80
WAN	68	3

To better distinguish different datasets, we assign each dataset a name. In this section, the datasets with name of XL, XXL and XXXL correspond to packages from PPDB. WN and WAN refer to WordNet and the University of South Florida Free Association Norms, respectively. Datasets with suffix “-Train” refer to the training sets with which we train the model, and those with suffix “-Test” refer to the testing sets. For link-based knowledge, we construct datasets named XL-Train, XL-Test, XXL-Train, XXL-Test, XXXL-Train, XXXL-Test, WN-Train, WN-Test, WAN-Train, WAN-Test. For preference-based knowledge, we have WANP-Train and WANP-Test datasets, which consist of about 263,000 and 10,000 triples respectively.

### 4.2 Evaluation Tasks and Metrics

To demonstrate that our model can efficiently encode the prior knowledge, we use the training sets to learn distributed word representation, and evaluate the models on testing sets. Since a training set and the corresponding testing set are extracted from the same knowledge source, a model that successfully encodes the prior knowledge in training set should be more consistent with the prior knowledge in testing set than the model trained without prior knowledge.

For link-based knowledge, we evaluate models by measuring the relatedness of word pairs in testing sets. The relatedness of a pair of words is calculated using the inner product of two word vectors. We assume the words in each pair in both training set and testing set are related with each other, hence a higher average relatedness of word pairs measured with our models indicates more consistency between the learned model and the testing link-based knowledge.

For preference-based knowledge, we evaluate models in the fashion of classification. For each triple (*w*_*s*_,*w*_*i*_,*w*_*j*_), if a learned model meets ${\vec{w}}_{s}\cdot {\vec{w}}_{i}>{\vec{w}}_{s}\cdot {\vec{w}}_{j}$, then the model correctly classifies the triple. In this way, we can use the accuracy of a model on a set of triples to measure the performance.

### 4.3 Parameter Settings

We use hierarchical softmax and negative sampling with 12 threads to train CBOW model as our baseline. In all of the following experiments, the dimension of each word vector is 500, and the window size is 15. The learning rate of SGD decreases linearly from a fixed initial value to 0.

It is obvious that a model will learn prior knowledge more sufficiently if running more SGD iterations in regularization threads. In experiments, the ratio of threads for learning *L*(*D*) and *R*(*D*,*P*) is set between 5:1 and 10:1 to get the best performance. That is, when five to ten threads are used for learning *L*(*D*), we will set one thread for learning *R*(*D*,*P*) with prior knowledge. Since the number of iterations in regularization threads exerts greater influence on the result, we focus on controlling the iterations of regularization threads instead of setting *λ* to balance *L*(*D*) and *R*(*D*,*P*) in Joint Optimization. We leave further discussion about the influence of iteration times in section 4.5.

There is a technical trick when training Euclidean Regularizer to improve performance. During each iteration of post optimization, we do not optimize each single link in isolation. Instead, for each word, we find all other words that link to this word and calculate the average vector of all word vectors as the center of all these words. We optimize the Euclidean distance between the target word and this center rather than the sum of distances between the target word and linked words. This will reduce the influence of noisy links in prior knowledge.

### 4.4 Experiment Results

With WN-Train dataset, we train different models with different optimization methods to compare the performance. The average relatedness achieved by various models are shown in Table 3. In this table, JO-SPR indicates Softmax Probability Regularizer trained by Joint Optimization, PO-SPR means Softmax Probability Regularizer trained by Post Optimization, and PO-ER means Euclidean Regularizer trained by Post Optimization.

**Table 3:** Average Relatedness of Different Methods.

Method	WN-Train	WN-Test
CBOW	0.084	0.083
JO-SPR	0.432	0.423
PO-SPR	0.153	0.153
PO-ER	**0.465**	**0.464**

From the table, we observe that, the models of JO-SPR and PO-ER achieve much higher consistency with the testing link-based knowledge than the original CBOW model. This indicates that, these models have successfully encoded the given knowledge into distributed word representations. It seems that PO-SPR is not so good as the other two models.

Afterwards, we use JO-SPR to train with different datasets to demonstrate that our model could learn from any set of link-based knowledge, from small sets such as WAN-Train, to large sets such as WN-Train and XXXL-Train. We also merge all training sets and train our model with the mixture of all link-based knowledge. The results of average relatedness on link-based knowledge are shown in Table 4. In this table, the column “Single Set” means that the JO-SPR model is trained using the training set of one dataset, and then evaluated on the training set itself or the corresponding testing set. The column “Multiple Sets” means that the JO-SPR model is trained using the link-based knowledge from all training sets and then evaluated on each dataset.

**Table 4:** Average Relatedness on Different Datasets.

Dataset	CBOW	Single Set	Multiple Sets
XL-Train	0.199	0.321	0.286
XL-Test	0.199	0.271	0.282
XXL-Train	0.141	0.254	0.218
XXL-Test	0.142	0.224	0.213
XXXL-Train	0.090	0.206	0.143
XXXL-Test	0.091	0.189	0.139
WN-Train	0.084	0.432	0.278
WN-Test	0.083	0.423	0.273
WAN-Train	0.161	0.241	0.192
WAN-Test	0.161	0.205	0.189

On each data set, the JO-SPR model significantly outperforms CBOW. Our model can learn link-based knowledge from all kinds of sources. On relative small datasets like XL-Train, the average relatedness of the JO-SPR model on XL-Test is apparently lower than that on XL-Train, as compared to large datasets. This indicates that the JO-SPR model exhibits more severe overfitting on small datasets, because the regularization threads will execute more iterations for a small prior knowledge. This issue will be discussed in detail in the next subsection.

On the other hand, when we combine knowledge from different sources together for learning a unique model, as shown in the column “Multiple Sets”, there will be much less overfitting. Our model performs nearly identical on both training sets and testing sets. Meanwhile, the performance on each dataset is consistently lower than that of training with only the corresponding training dataset. The reasons are: (1) datasets from different sources are not completely consistent with each other; and (2) the average number of iterations for each link is diluted so the learning process might be not so sufficient.

**Fig 4:**
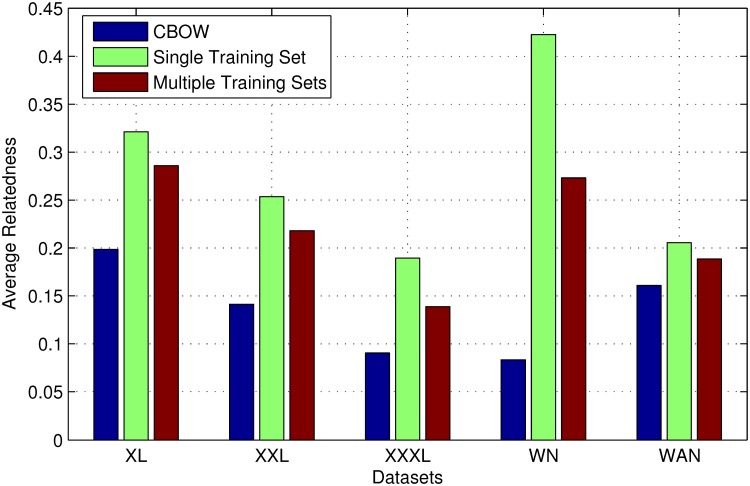
Average relatedness.

To better visualize the performance on different datasets, the results on testing sets are also shown in Fig. 4. The improvement of our model on average relatedness depends on the datasets. Especially on WN-Test, the average relatedness of our model is about 5 times as compared to the original CBOW model, which indicates that the links in WordNet is more consistent inherently. It is not surprising because WordNet is built by several experts with more stable criteria and thus contains less noise.

To learn preference-based knowledge, we train Negative Sampling Regularizer with Joint Optimization (JO-NSR) and Margin Regularizer with Post Optimization (PO-MR). The results of preference classification accuracy are shown in Table 5. JO-NSR shows great improvement in accuracy as compared to the original CBOW model, and PO-MR can fit the data with significant accuracy. In Section 4.6, we will demonstrate that PO-MR model has drawback of overfitting.

**Table 5:** Preference Classification Precision of Different Methods.

Method	WANP-Train	WANP-Test
CBOW	0.628	0.628
JO-NSR	0.746	0.717
PO-MR	0.942	0.914

### 4.5 Influence of Iteration Times in Joint Optimization

For the JO scheme, the number of iterations in SGD directly influences the consistency between learned vectors and prior knowledge. We take two strategies to control the number of iterations and explore its influences.

- Change the numbers of threads for learning from raw corpora and prior knowledge, respectively. Since the overall computation of learning from raw corpora is almost constant, hence creating more threads for regularization will lead to more regularization iterations.
- Let the threads for learning from prior knowledge sleep for a short period (typically from 10 ms to 500 ms) every 1000 iterations. This strategy can reduce the number of regularization iterations arbitrarily.

Considering that different datasets are of different scale, it is more reasonable to control the relative iteration times rather than absolute iteration times. Take JO-SPR with link-based knowledge for an example, we control the number of iterations divided by the number of links, i.e., the iteration times per link, as the independent variable.

**Fig 5:**
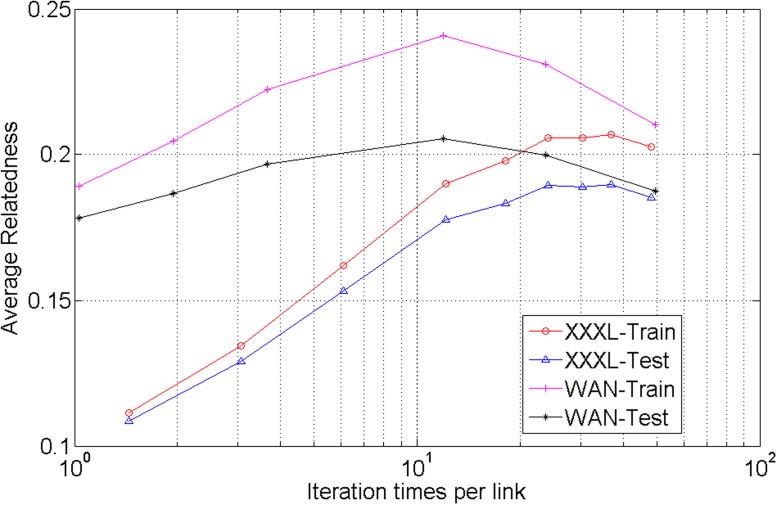
Influence of iterations per Link.

We train with link-based knowledge from datasets of XXXL and WAN separately and evaluate the average relatedness of both training set and testing set of XXXL and WAN. The results are shown in Fig. 5. As the increase of iteration times per link, the average relatedness increases at first, then decreases after the peak is achieved. The reason is that, too many iteration times will lead to overfitting on training sets and make the average relatedness on testing sets drop.

Another observation is that higher average relatedness on training set is accompanied by larger divergence in average relatedness between training set and testing set. It is important to realize trade-offs between fitting the knowledge and ensuring the generalization. This is a common issue that should be taken into consideration for most statistical learning algorithms.

### 4.6 Semantic Relatedness Ranking

In the previous subsections, we have demonstrated that our models can successfully incorporate the prior knowledge into distributed word representations. However, the previous evaluation is not enough, because the goal of incorporating prior knowledge is to obtain high quality word representations rather than just fitting knowledge bases. Therefore, we further investigate the performance of word representations for semantic relatedness ranking after incorporating prior knowledge.

Semantic relatedness ranking is a classical task to evaluate quality of word representations. The evaluation dataset usually contains a list of word pairs. For each word pair, human annotators are asked to determine how semantically related they are, and the annotations are considered as gold standard. Given the evaluation dataset, word representation models can also compute the semantic relatedness of these word pairs. Then the Spearman’s rank correlation coefficient (Spearman’s *ρ*) between human and model rankings could be used to measure the quality of word representations.

Wordsim-353 [14] is a widely-used dataset for semantic relatedness ranking. It has been used to evaluate many different word representation systems. Word representation with multiple prototypes [15] aims to build multiple distinct vectors for all senses of a word, which is expected to be more discriminative than traditional word representation methods. Tiered clustering [16] proposes a mixture model to derive multiple prototype representations for word senses, which can also be used to measure word relatedness. The two methods both achieve a score of about 0.77. WN30G [17] utilizes WordNet to determine the semantic relatedness between word pairs and achieves a Spearman’s *ρ* of 0.66. ESA [18] is another knowledge-based method that learns distributional representations of words with the favor of Wikipedia and achieves a score of 0.75.

In Table 6, we list the Spearman’s *ρ* of some previous work and our models. For our models, we also consider mixing various knowledge sources. The mixing method is straightforward, i.e., we incorporate one type of knowledge in joint optimization and incorporate another type of knowledge in post optimization. In Table 6 we use JO-SPR and PO-ER to mix two types of knowledge, including XXXL + WN and WAN + WN.

**Table 6:** Spearman’s *ρ* on Wordsim-353 Dataset.

Model	Knowledge	WS353
Multiple Prototypes	-	0.770
Tiered Clustering	-	0.769
WN30G	WordNet	0.660
ESA	Wikipedia	0.750
CBOW	-	0.734
JO-SPR	XL	0.756
JO-SPR	XXL	0.754
JO-SPR	XXXL	0.765
JO-SPR	WN	0.746
JO-SPR	WAN	0.758
PO-ER	WN	0.764
JO-NSR	WANP	0.734
PO-MR	WANP	0.707
JO-SPR, PO-ER	XXXL, WN	**0.778**
JO-SPR, PO-ER	WAN, WN	**0.784**

From Table 6 we observe that JO-SPR combined with PO-ER achieves the best Spearman’s *ρ* of 0.784, outperforming both pure unsupervised learning method and traditional knowledge-based methods. From the table, we conclude that: (1) Link-based knowledge is helpful to enhance the quality of distributed word representations, and different datasets bring improvements in different degrees. (2) Preference-based knowledge does not directly contribute to improvements in this task. Perhaps information depicted by preference-based knowledge is too detailed, and cannot have a good coverage over the evaluation dataset. (3) It is acceptable that JO-NSR learns preference-based knowledge without hurting the overall quality of word representation. But for PO-MR the strong tendency of fitting knowledge is harmful in the task due to overfitting. (4) Better representations can be produced by mixing various knowledge sources with different methods into one single model. According to our experiments, the combinations of JO-SPR trained with XXXL or WAN dataset and PO-ER trained with WN dataset beat all other models trained with single datasets. This indicates that, appropriate combinations of knowledge sources may significantly improve the quality of distributed word representations.

In experiments, we have tried to decrease the ratio of threads for learning *L*(*D*) and *R*(*D*,*P*) to emphasize the role of prior knowledge. We find that when the thread ratio comes to 9:6, the performance on wordsim-353 reduces to less than 0.4. The reason may be that, the coverage of prior knowledge is not extensive enough to provide sufficient information for learning good representation itself, and thus can only work as supplementary information.

We also analyze the variances of word-pair ranking lists obtained by CBOW and our model of JO-SPR (XXXL) + PPO-ER (WN). We regard the human-annotated rankings of word pairs as gold standard. Our model makes 193 out of 353 word pairs closer to the gold standard rankings. If we only consider the 123 word pairs with the most significant ranking variances (the ranking change is over 35, i.e., about 10% of the whole list), there are 74 of them become closer to gold standard rankings.

The incorporation of prior knowledge may also bring some noise. This noise hurts word representation learning by either making some related words far from each other, or making some unrelated words close to each other. Take the word pair *lad* and *brother* for example. In gold standard, the ranking of the word pair is 262, and CBOW ranks the word pair in 280, but our model ranks the pair in 36. The reason is that, the knowledge in PPDB-XXXL believes the two words are related to each other, and the word *lad* is infrequent in our text corpus. Hence our model tends to learn word representation of *lad* according to the prior knowledge. Take another word pair *tennis* and *racket* for example. The rankings by gold standard, CBOW and our model are 87, 47 and 167. Our model underestimate the relatedness of the two words simply because the prior knowledge does not mention any relatedness information for the word pair.

Note that, the rankings in gold standard are not always reliable. For example, the word pair *precedent* and *antecedent* is ranked in 192 by gold standard. In fact, the two words are related to each other. Since both words are infrequent and their word vectors cannot be sufficiently learned from text corpus, hence CBOW only ranks them in 187. Due to the favor of prior knowledge, our model successfully identifies their relatedness and ranks them in 30.

In summary, the prior knowledge may either enhance or conflict with word representations learned from text corpus. We should find appropriate prior knowledge to incorporate in learning word representations.

## Related Work

### 5.1 Word Representation

There have been various approaches for word representation, including one-hot representation, distributional representation and distributed representation.

In one-hot representation, the length of word vectors is identical to the size of the vocabulary. For the vector of a specific word, only one dimension is on, indicating the corresponding word. The representation is usually used as the basis of document representation, e.g., bag-of-words model [1], which usually suffers from sparsity issue.

As mentioned in the introduction section, distributional representation assumes similar words tend to share similar contextual distributions [2]. Since simple co-occurrence-based distributional representation does not work well, techniques such as reweighting, smoothing and dimension reduction have been proposed. Typical models include self-organizing semantic map [19–21] and latent semantic analysis (LSA) [22]. Besides, clustering techniques based on contextual information have also been proposed for distributional representation, such as Brown Clustering [23].

Most methods for distributional representation are unsupervised. It have been empirically verified that prediction-based distributed representation generally outperforms distributional representation [24, 25]. Distributed representation estimates word vectors so as to maximize the probability of the contexts given target word. Statistical language models based on neural networks, as the representative distributed representation methods, have been widely used in various applications including POS-tagging, entity recognition, syntactic parsing and semantic role labeling [3–5, 24, 26–29]. The characteristics of *word2vec* for modeling implicit relations of words have also been used to extract semantic hierarchies of words from plain texts [30].

Computational complexity of above-mentioned distributed representation models are usually high, which make these models not feasible for large-scale corpora in big data era. To address this issue, Continuous Bag-of-Words Model (CBOW) and Continuous Skip-gram Model [6, 11], which discard non-linear hidden layer and manage to learn large-scale corpora efficiently. To our knowledge, there has been few work on incorporating prior knowledge into distributed word representation.

### 5.2 Learning with Prior Knowledge

Prior knowledge has been considered in model learning in many tasks. The most related one is latent topic modeling with prior knowledge. For example, document networks are considered as a regularizer in topic models [31]. Similarly, Dirichlet Forest is employed to encode Must-Links and Cannot-Links among words given by domain knowledge for topic modeling [32]. Moreover, First-Order Logic is used to encode domain knowledge for topic modeling [33].

In this paper, we also adopt regularization-based method to incorporate prior knowledge for distributed word representation. Different from the version in topic modeling, we propose specific objective functions and optimization techniques in consideration of the characteristics of word representation models. In experiments we have demonstrate the effectiveness of our methods.

The word representation model in [24] provides semi-supervised framework to learn word representation. However the supervision comes from a small amount of labeled text. This type of knowledge is indirect, because it only contains the expected function or usage of words, rather than word meanings and relations among words.

## Conclusion and Future work

In this paper, we propose a unified framework to incorporate prior knowledge into distributed word representation. It is expected that, prior knowledge can compensate for information that is not contained in text corpora. We consider two types of knowledge, i.e., link-based knowledge and preference-based knowledge. For the two types of knowledge, we propose specific objective functions and incorporate them into word representation learning as a regularizer. We further present joint optimization and post optimization for parameter estimation.

In experiments, we demonstrate that this is a promising way to improve the quality of distributed word representation. Considering that accessible corpora may be insufficient both in amount and quality for representation learning of all words, especially in some domain-specific situations, utilization of prior knowledge is a practical solution to obtain better word representation and benefit other NLP tasks. Moreover, this work is a general framework, which can be easily extended to other distributed representation methods.

Essentially, word representation learning is a type of representation learning. What we are trying to emphasize is that, prior knowledge of words can really help word representation models to better capture semantic meanings of words. As for a specific NLP task, such as relation inference, annotated data should be the most direct knowledge to leverage. Word representations learned with indirect prior knowledge such as WordNet are expected to provide better initialization for further optimization.

The methods proposed in this paper is an encouraging start of learning distributed word representation with prior knowledge. There are many challenging topics for further exploration, and we list some as future work:

- Types of prior knowledge. In this paper, we mainly focus on incorporating word knowledge. We can explore more complicated prior knowledge, such as the world knowledge in Wikipedia. Complicated entity relations in large-scale knowledge graphs may provide more useful information for word representation [34, 35].
- Representation of prior knowledge. In this paper we represent prior knowledge into two types. We can also design more expressive representation scheme, such as the first-order logic.
- Word representation methods. We may consider incorporate prior knowledge into other distributed word representation methods. For example, distributed representation of multiple word prototypes [15] can also be combined with prior knowledge. With synsets in WordNet, we can learn multiple word prototypes and disambiguate word senses in corpora simultaneously.
